# Telomere shortening may be associated with human keloids

**DOI:** 10.1186/1471-2350-10-110

**Published:** 2009-10-28

**Authors:** Bruna De Felice, Robert R Wilson, Massimo Nacca

**Affiliations:** 1 Department of Life Sciences, University of Naples II, Via Vivaldi 43, 81100 Caserta, Italy

## Abstract

**Background:**

Keloids are benign skin tumors that are the effect of a dysregulated wound-healing process in genetically predisposed patients. They are inherited with an autosomal dominant mode with incomplete clinical penetrance and variable expression. Keloids are characterized by formation of excess scar tissue beyond the boundaries of the wound. The exact etiology is still unknown and there is currently no appropriate treatment for keloid disease.

**Methods:**

We analyzed sample tissues were obtained from 20 patients with keloid skin lesions and normal skin was obtained from 20 healthy donors. The telomeres were measured by Terminal Restriction Fragment (TRF) analysis and Real-Time PCR assay. Quantitative Real-Time RT-PCR analysis of hTERT gene expression was performed and intracellular ROS generation was measured.

**Results:**

In this study, we determined whether telomeric shortening and the expression of human telomerase reverse transcriptase (hTERT) occurs in keloid patients. Using Terminal Restriction Fragment (TRF) analysis and Real-Time PCR assay, we detected a significant telomere shortening of 30% in keloid specimens compared to normal skin. Using quantitative Real-Time RT-PCR, telomerase activity was found absent in the keloid tissues. Moreover, an increase in ROS generation was detected in fibroblasts cell cultures from keloid specimens as more time elapsed compared to fibroblasts from normal skin.

**Conclusion:**

Telomere shortening has been reported in several metabolic and cardiovascular diseases. We found that telomere shortening can also be associated with human keloids. Chronic oxidative stress plays a major role in the pathophysiology of several chronic inflammatory diseases. Here we found increased ROS generation in fibroblasts from keloid fibroblasts cell cultures when compared to normal skin fibroblasts. Hence we conclude that oxidative stress might be an important modulator of telomere loss in keloid because of the absence of active telomerase that counteracts telomere shortening.

## Background

Keloids (OMIM 148100) are benign skin tumors occurring during wound healing in genetically predisposed patients. The pattern of inheritance observed in 14 pedigrees with familial keloids has been consistent with an autosomal dominant mode with incomplete clinical penetrance and variable expression [1]. Keloids are characterized by the proliferation of dermal fibroblasts, overproduction of extracellular matrix components (ECM), an increased infiltration of inflammatory cells including lymphocytes, mast cells (MCs) and macrophages. Keloids spread to invade normal skin beyond the boundaries of the original wound and do not regress spontaneously [2]. Previously, we showed that keloids differed from what we found in hypertrophic scars in that p53 down regulation, together with the increased ΔNp63 expression, could contribute to keloid development by means of accumulation of continuously proliferating cells [3]; we also found a significant increase in ROS (reactive oxygen species) generation in keloid fibroblasts, a phenomenon that probably relates to the inflammatory and oxidative stress status of the disease [3]. It is reasonable to believe that the lack of effective therapies is due to an insufficient understanding of the disease pathology. This is partially because the study of keloid biology is hindered by the lack of a well-established animal model for the disease. Telomeres, the TTAGGG tandem repeats at chromosomal ends, become progressively shorter with each replication of somatic cells. Telomerase is a reverse transcriptase that synthesizes the telomeric sequence, which prevents telomere shortening and thereby prolongs the lifespan of cells. Activation of telomerase is determined by the expression of the telomerase reverse transcriptase (hTERT) mRNA. This enzyme, which is known to play a major role in telomeres maintenance, is present in the majority of cancer and germ cells as well as in normal endothelium, while enzyme activity is absent in most normal human somatic cells [4]. It has reported that oxidative stress is an important modulator of telomere loss and that telomere-driven replicative senescence is primarily a stress response [5]. Telomere shortening has been found in several inflammatory diseases such as vascular diseases, Type 2 Diabetes, Fanconi anaemia, Ataxia Telangiectasia [6-9].

There are no available data on telomere length in human keloids, and we hypothesized that keloid patients would demonstrate shorter telomeres compared with control subjects and that this would be directly related to markers of oxidative DNA damage.

Thus, the purpose of this study was to compare the telomere length of keloids patients and control subjects; moreover, we investigated the possible role of telomerase in keloids determining the expression of human telomerase reverse transcriptase (hTERT) mRNA, which is the minimal component needed for telomerase activity. This is the first report, to our knowledge, showing telomere shortening in human keloids.

## Results

**Figure 1:**
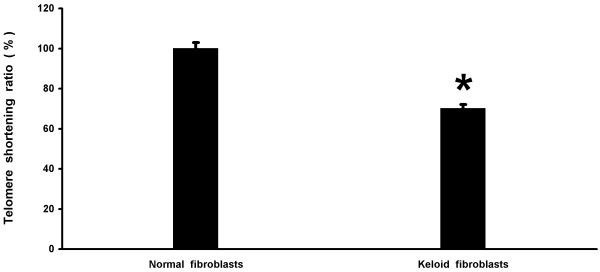
**Quantitative estimation of the telomeres shortening rate (%) between normal skin and keloid**. Telomere shortening rate in normal skin and keloid specimens. Data are mean (± SE) of telomere lengths from the 20 controls and the 20 patients with keloids measured in triplicate. The asterisk indicates a significant difference between normal skin and keloids with *P < 0.05.

**Figure 2:**
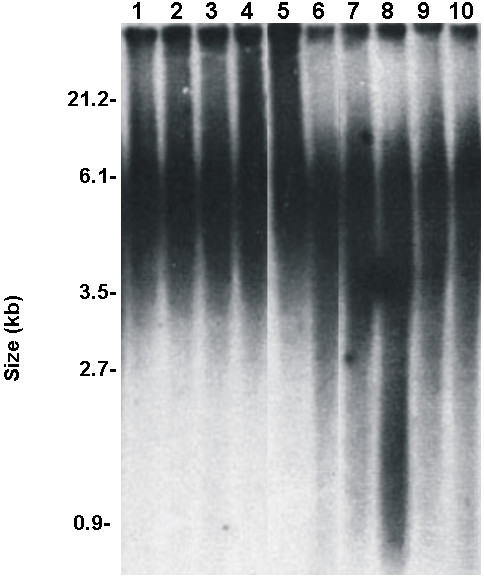
**Telomere Southern blot of normal skin and keloid specimens**. Terminal restriction fragment length (TRF) was determined by Southern blot analysis and a subsequent densitometric analysis. Equivalent amounts of genomic DNA were loaded in each lane. (1-5) TRF length of normal skin and (6-10) keloid specimens.

**Figure 3:**
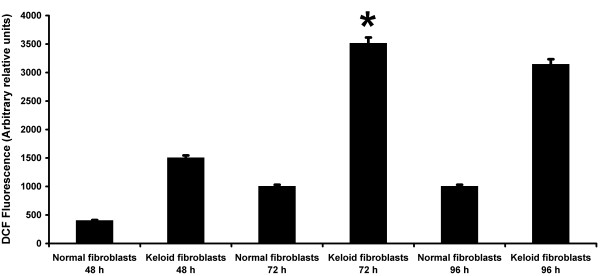
**ROS generation**. Intracellular ROS generation was determined by measuring the fluorescence intensity of the oxidation-sensitive fluorescein DCFH-DA of fibroblasts cell culture from the 20 normal and keloid specimens respectively in time course experiments at 48, 72 and 96 hours. The histogram shows the mean values ± SD of ROS generation obtained by analysis of three separate experiments. * P < 0.05

**Figure 4:**
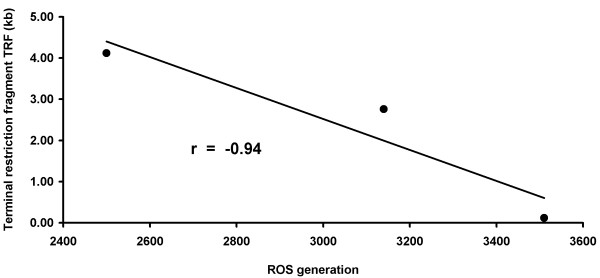
**ROS and telomere length correlation**. Relationship between ROS generation and telomere length in keloids. Data shown were obtained from 20 keloid patients (*r *= -0.94) P < 0.01.

For measuring telomere length, Terminal Restriction Fragment (TRF) analysis and Real-Time PCR assay were both used in all samples. Among the 20 keloid patients, the mean (+/- SD) telomere length was significantly lower in the patients with keloids (4.12 ± 0.2 kb) compared with the 20 control subjects (6.070 ± 0.6 kb) (*P < 0. 05) (Figure 1 and 2). Therefore, it was reasonable to hypothesize that telomerase, the reverse transcriptase that elongates telomeres through the de novo synthesis of TTAGGG repeats, was more inactive in keloid patients. To investigate the possible role of telomerase activity in human keloid, we analyzed hTERT expression level, by a quantitative RT-PCR assay using primer pairs specific for hTERT mRNA in either keloid skin and normal skin. hTERT expression was assayed in the tip and in the edge of the keloid specimens and in the adjacent normal skin, but the level was undetectable. hTERT expression was assayed in the tip and in the edge of the normal skin specimens too and no significant expression was detected (data not shown). Here, we made fibroblasts cell cultures from the 20 keloid and normal skin specimens used in this study. An increase in ROS generation was detected in fibroblasts cell cultures from keloid specimens with elapsing time, peaking at 72 hours, compared to fibroblasts from normal skin (Figure 3). Decreased telomere lengths were significantly correlated with ROS levels (*r *= -0.94) in fibroblasts from keloid specimens (Figure 4).

## Discussion

Shortening of telomeres has been reported to be present in patients with inherited respiratory chain disorders [10], Down's syndrome [11], vascular dementia [12] and ataxia-telangiectasia [13]. Recent studies have also shown that telomere shortening can be a biomarker of premature cell senescence in vascular diseases and metabolic disorders [14-16]. Our study is the first to demonstrate a shortened TRF length in patients with keloids. It is still controversial if there is a correlation between telomerase activity and hTERT expression. In humans, telomerase activity is detected in germ cells and in nearly all malignant tumor cells. Telomerase is not detected in the majority of somatic cells except for in a few somatic cells such as lymphocytes and endometrium of the uterus [17]. hTERT, the core component of human telomerase, is detected only in cells and tissues positive for telomerase activity, but it is not present in normal somatic cells lacking telomerase activity. It was recently reported that several normal tissues lack telomerase activity despite expressing hTERT mRNA [18,19]. Furthermore, the cellular defending function of telomerase has been correlated to an increased apoptosis resistance and to DNA damage repair. It has been shown that oxidative stress induces the depletion of human telomerase reverse transcriptase hTERT from the nucleus through the nuclear pores into the cytosol [20,21].

Telomere length and attrition rate may be prognostic for several forms of cardiovascular diseases, particularly if oxidative stress outlines in the development of these diseases [22]. Telomeric DNA is more sensitive to oxidative damage than the whole genome; indeed telomeres are particularly prone to damage at the GGG sequence compared with the rest of chromosomal DNA and relatively modest degrees of oxidative DNA damage could be expressed as substantial telomere attrition. Interestingly, patients with ROS production due to mitochondrial DNA defects also had shortened telomeres and oxidative stress caused enhanced telomere shortening and loss of function in Fanconi Anaemia cells [23];moreover, cells from patients with ataxia-telangiectasia show enhanced sensitivity of telomeric DNA to oxidative stress [24] Recent studies have demonstrated that chronic oxidative stress interferes with telomere maintenance at two levels: it increases the basal rate of telomere shortening by induction of telomeric DNA damage, as shown before in telomerase-negative fibroblasts, and it prevents telomerase from counteracting telomere shortening by inducing its export from the nucleus and to mitochondria; telomerase can be excluded from the nucleus in parallel with ROS generation [25]. In a previous study we found a significant increase in ROS (reactive oxygen species) generation in fibroblast cell cultures from keloid patients compared to fibroblasts cell cultures from normal patients, a phenomenon that probably relates to the inflammatory and oxidative stress status of the disease [3]. These results have been confirmed here, since a significant increase in ROS generation was detected in keloid fibroblasts cell cultures from keloid specimens when compared to normal fibroblasts.

It is possible to hypothesize that oxidative stress may modify TRF length in keloids; otherwise a diminished TRF length could take place for telomere attrition due to an increased turnover and chronic activation of inflammatory cells. Telomere attrition can be an independent keloid risk factor or a consequence of the disease, since accelerated shortening of telomere length could simply be a surrogate for the chronic oxidative stress and/or inflammation.

We propose that, such as in other inflammatory diseases, oxidative stress might contribute to telomere shortening in human keloids.

## Conclusion

Our study, for the first time, reported that there is an association between telomere length and keloids, with age and gender adjusted; however, a larger-scale study is required to explore the potential role of environmental (oxidative and more) stresses in the mechanism of telomere shortening in human keloids.

## Methods

### Keloid samples and DNA extraction

Since telomere length is inversely related to chronological age and was greater in women than in men, the effects of age and sex were controlled to obtain an absolute relationship between keloids and telomere length. All subjects (20 patients with keloids and 20 without) were males, within an age range of 25-35 years, and the age of the scar was between 10-12 months old (Table 1). Fresh tissues were obtained from patients with keloid skin lesions and normal skin was obtained from healthy donors.

**Table 1:** Clinical characteristics of the study subjects

**Parameters**	**Control** **(*n *= 20)**	**Patients with keloids (*n *= 20)**
Age (years)	25-35	25-35
Gender	male	Male
Age of the scar (months)		10-12
Mean (± SD) Telomere kb Length. * *P *< 0.05	6.070 ± 0.6 kb	4.12 ± 0.2

The study was performed with the patients informed consent and approved by the Ethic Committee of the University of Naples (Italy) in compliance with the Helsinki Declaration.

Genomic DNA was prepared from skin specimens using DNA Isolation Kit for Cells and Tissues (Roche, Mannheim, Germany).

### TRF length analysis

The telomeres were measured using a TeloTAGGG Telomere Length Assay (Roche, Mannheim, Germany) following the manufacturer's recommendations, and also measured using a Real-Time PCR method, as described by Cawthon [26].

### Real-Time Quantitative RT-PCR for hTERT

Total RNA from skin specimen and quantitative Real-Time RT-PCR analysis of hTERT gene expression was performed as already reported [27].

### Cell cultures

Fibroblasts from keloids and normal skin were isolated from samples following the method established by Lim et al [28].

### Measurement of ROS generation

The intracellular ROS generation was measured by incubating the cells in the presence of 2',7'-dichlorodihydrofluorescein diacetate (DCFH-DA) (Calbiochem). DCFH-DA is a stable, non-fluorescence molecule able to cross the cell membranes and is hydrolyzed by intracellular esterases to the non-fluorescent probe DCFH, that in turn is rapidly oxidized in the presence of peroxides to the highly fluorescent 2',7'-dichlorofluorescein (DCF). Cells (3.5 × 10^5^/mL) were loaded with 1 μM DCFH-DA for 30 min at 37°C in standard medium, whose composition in mM was: NaCl 138, KCl 2.7, CaCl2 1.2, MgCl2 1.2, phosphate-buffered saline (PBS) 10, glucose,10, pH 7.4. After the loading period, cells were washed twice with phosphate buffered medium before the experiment was performed. Acquisition data were obtained using a fluorescent sensitive camera Cool Snap (Media Cybernetics Inc., Silver Spring, MD, USA) and cells were evidenced by a complex software Image-Pro Plus 4.5 (Media Cybernetics Inc.) by which areas were examined.

### Statistical analysis

All results shown are mean +/- SD of at least three separate experiments, measuring each parameter by triplicate (n = 3). Statistical significant differences were tested by one way analysis of variance (ANOVA), and, when the *F *value was significant, by Student-Newman-Keul' s test. *P *value less than 0.05 (*) was considered statistically significant. In addition, we computed Pearson correlation coefficient to describe associations between measures.

## Competing interests

The authors declare that they have no competing interests.

## Authors' contributions

BDF carried out this study, supervised the project and drafted the manuscript. MN provided experimental assistance. RRW provided analytic support and data assistance. All authors have read and approved this final manuscript.

## Pre-publication history

The pre-publication history for this paper can be accessed here:


